# Optimized ChIP-exo for mammalian cells and patterned sequencing flow cells

**DOI:** 10.1093/g3journal/jkaf270

**Published:** 2025-11-07

**Authors:** Daniela Q James, Sohini Mukherjee, C Caiden Cannon, Shaun Mahony

**Affiliations:** Center for Eukaryotic Gene Regulation, Department of Biochemistry and Molecular Biology, The Pennsylvania State University, University Park, PA 16802, United States; Center for Eukaryotic Gene Regulation, Department of Biochemistry and Molecular Biology, The Pennsylvania State University, University Park, PA 16802, United States; Center for Eukaryotic Gene Regulation, Department of Biochemistry and Molecular Biology, The Pennsylvania State University, University Park, PA 16802, United States; Center for Eukaryotic Gene Regulation, Department of Biochemistry and Molecular Biology, The Pennsylvania State University, University Park, PA 16802, United States

**Keywords:** ChIP-exo, protocols, sonication, chromatin, chromatin immunoprecipitation, NGS, NextSeq 2000, CTCF

## Abstract

By combining chromatin immunoprecipitation (ChIP) with an exonuclease digestion of protein-bound DNA fragments, ChIP-exo characterizes genome-wide protein–DNA interactions at near basepair resolution. However, the widespread adoption of ChIP-exo has been hindered by several technical challenges, including lengthy protocols, the need for multiple custom reactions, and incompatibilities with recent Illumina sequencing platforms. To address these barriers, we systematically optimized and adapted the ChIP-exo library construction protocol for the unique requirements of mammalian cells and current sequencing technologies. We introduce a mammalian-optimized ChIP-exo (MO-ChIP-exo) protocol that builds upon previous ChIP-exo protocols with systematic optimization of crosslinking, harvesting, and library construction. We validate MO-ChIP-exo by comparing it with previously published ChIP-exo protocols and demonstrate its adaptability to both suspension (K562) and adherent (HepG2, mESC) cell lines. This improved protocol provides a more robust and efficient method for generating high-quality ChIP-exo libraries from mammalian cells.

## Introduction

Characterizing the genome-wide binding locations of transcription factors and other regulatory proteins is crucial for understanding complex cellular functions. Chromatin immunoprecipitation (ChIP) combined with high-throughput sequencing (ChIP-seq) (Albert et al. 2007; Johnson et al. 2007) emerged as the main method to identify genome-wide patterns of protein–DNA binding in a wide variety of species and cell types (Dunham et al. 2012; Moore et al. 2020). While newer approaches such as CUT&RUN (Skene and Henikoff 2017) and CUT&Tag (Kaya-Okur et al. 2019) have provided valuable alternatives, they are most popularly applied to characterize histone modifications (Abbasova et al. 2025). ChIP-seq remains the preferred method to study mammalian transcription factor binding. ChIP-exo greatly improved upon the resolution of ChIP-seq by incorporating an exonuclease to digest immunoprecipitated DNA fragments in a strand-specific direction until blocked by a crosslinked protein (Rhee and Pugh 2011, 2012). As a result, ChIP-exo yields sharper protein-bound fragments and lower background than ChIP-seq, enabling the identification of binding events at near basepair resolution. In yeast, ChIP-exo was applied to study transcription factor binding (Rhee and Pugh 2011) and to characterize the organization of individual histones (Rhee et al. 2014). Most notably, ChIP-exo was used to characterize the genome-wide architecture of all yeast chromatin-associated proteins in the Yeast Epigenome Project (Rossi et al. 2021). It has also been applied to screen a large cohort of monoclonal antibodies generated by the Protein Capture Reagents Program (Lai et al. 2021).

Despite its advantages, the widespread adoption of ChIP-exo has been hindered by several technical challenges, including lengthy protocols, the need for multiple custom reactions, a lack of quality control checkpoints, and higher costs. Previous efforts have attempted to address some of these issues. The original protocol was initially adapted from the SOLiD (ChIP-exo 1.0) to the Illumina sequencing platform (ChIP-exo 1.1) (Serandour et al. 2013; Yen et al. 2013). The ChIP-nexus protocol adapted ChIP-exo to incorporate more efficient adapter ligation and library amplification strategies with single-end sequencing (He et al. 2015). Most recently, Rossi et al. (2018) presented a simplified protocol (ChIP-exo 5.0), which greatly reduced the number of enzymatic steps. However, these efforts were tailored to the technologies available at the time, including random patterned flow cells, combinatorial index IDs for multiplexing, and gel excisions for size selection. The advent of newer Illumina platforms that use patterned flow cells and exclusion amplification clustering (eg the NextSeq 2000) has revealed and potentially exacerbated issues in the ChIP-exo 5.0 protocol. Furthermore, previous protocols were primarily optimized to target yeast or *Drosophila* and have not been systematically optimized for mammalian cells.

These challenges prompted us to reevaluate and adapt the ChIP-exo library construction protocol, specifically considering recent advancements in sequencing technology and the unique requirements of mammalian cells. In this manuscript, we highlight key protocol modifications for generating ChIP-exo libraries from mammalian cell lines. We present a series of improvements developed through the systematic exploration of multiple conditions for cell harvesting and library construction, building upon the foundation of the ChIP-exo 5.0 protocol.

## Materials and methods

### Cell culture, crosslinking, and harvest

K562 (CCL-243) and HepG2 (HB-8065) cells were obtained from the American Type Culture Collection handled and subcultured following the manufacturer’s recommendations. A17 mES cells were cultured as previously described (Bulajić et al. 2020). The inducible cassette exchange system (Iacovino et al. 2011) was used to generate A17 mES cells containing a doxycycline-inducible copy of the FOXA1 DNA-binding domain (DBD) with a 3× FLAG tag. For the FOXA1-DBD experiments, the transgene was induced with 3 μg/mL of doxycycline for 24 h before crosslinking.

K562 suspension cells were counted and directly crosslinked in media or pelleted and resuspended for crosslinking in phosphate-buffered saline (PBS). Adherent cells were washed with PBS, trypsinized, quenched, and resuspended in media or PBS for counting and crosslinking. All cell counts were performed with a trypan blue dye exclusion test using a hemocytometer. For mammalian-optimized ChIP-exo (MO-ChIP-exo) protocol, 10 min of crosslinking with 1% formaldehyde was quenched with a 3 M Tris pH 7.8. Variations of the crosslinking time, formaldehyde concentration, and quencher were used as reported. Crosslinked cells were washed with PBS + 1× CPI and flash frozen in liquid nitrogen. A detailed crosslinking and harvest protocol is provided in Supplementary Methods.

### MO-ChIP-exo sonication and library construction

Crosslinked cell pellets (10-M cells per reaction) were subjected to cell and nuclear lysis. Lysates were resuspended in PBS + 1× CPI for sonication in a Diagenode Bioruptor Pico. The “Go & Shear” setting was used with the validated parameters on the “Easy Mode” for 30″ On/Off with increasing cycles (1 to 11 as described). An aliquot of 15 μL (∼0.5-M cells) was used to reverse crosslink and purify the DNA for a sonication assessment using the Agilent Tape Station D5000 kit. The sonicated crosslinked chromatin was precleared before ChIP using high-speed centrifugation (14k rpm at 4 °C for 15 min). ChIP antibodies (anti-CTCF [Sigma 07-729], anti-USF1-1B8 [DSHB USF1-1B8-6/13/19], anti-FLAG [Sigma, F1804], IgG [Sigma i5006], anti-GATA1 [Santa Cruz Biotechnology SC-266], anti-FOXA1 [Santa Cruz Biotechnology SC-101058], and Anti-SOX2 [R&D AF2018]) were preconjugated to 50 μL Protein A or G Dynabeads (Invitrogen) before an overnight incubation with the antibody of interest at 4 °C. Bead-bound chromatin was washed and subjected to simultaneous end repair and A-tailing with a subsequent TA ligation of the first high-performance liquid chromatography (HPLC)-purified adapter using the NEBNext Ultra II DNA Library Prep Kit. After washes, a fill-in reaction was performed using Phi29 DNA Polymerase (NEB). This reaction was followed by another wash and lambda exonuclease (NEB) digestion. Reverse crosslinking was performed overnight to release the DNA. Released DNA was purified using Mag-Bind TotalPure NGS beads (Omega Bio-Tek) before ligating the second HPLC-purified adapter using T4 DNA ligase (Qiagen). A DNA cleanup was performed using Mag-Bind TotalPure NGS beads before PCR enrichment for 12 cycles. This was followed by size selection using SPRI select beads (Beckman Coulter) and an additional DNA cleanup to remove excess adapters. Completed libraries were analyzed in the Tape Station using High Sensitivity D5000 ScreenTape and Reagents. Finally, libraries were individually quantified through qPCR using NEBNext Library Quantification Kit, For Illumina (NEB) in an ABI StepOne Plus Real-Time PCR System. A detailed MO-ChIP-exo protocol is provided in Supplementary Methods and has been deposited in the protocols.io repository (doi:10.17504/protocols.io.3byl46jjjgo5/v1).

### Input assessment using ChIP-qPCR and western blot

The input and CTCF-ChIPed DNA from different crosslinking conditions were quantified using a Qubit 1× dsDNA HS Assay Kit (Thermo Scientific) and used as templates for qPCR comparative enrichment using the % input method. Anti-CTCF (Sigma 07-729) was used for the ChIP. The NEB Luna Universal qPCR Master Mix was used with primers targeting the KLK locus (KLK2, KLK5) and a CTCF unbound region (WNT2) (Khoury et al. 2020). CTCF-ChIPed DNA was normalized to input and each condition was quantified in triplicate. To assess CTCF protein abundance, western blot was used. To prepare lysates, K562 cells were washed in ice-cold PBS + 1×CPI and collected by centrifugation. The cell pellet was lysed in 1.5× Laemmli buffer and boiled at 95 °C for 5 to 10 min. After centrifugation at 14k rpm for 10 min, the supernatant was collected and stored at −20 °C. Lysates were reboiled for 3 min upon thawing and separated through SDS–PAGE using 8% polyacrylamide gels. Gels were transferred using the Trans-Blot Turbo Transfer System and RTA Mini 0.2-μm Nitrocellulose Transfer Kit (Biorad). Blots were blocked in 5% milk in TBS-T for 1 h at room temperature. CTCF (G-8) HRP (Santa Cruz Biotechnology sc-271474) was diluted 1:1,000 in 5% milk in TBS-T and incubated overnight with gentle rocking at 4 °C. TBS-T washes were performed before developing with Pierce ECL Western Blotting Substrate (Life Technologies) using a ChemiDoc Imaging System (Biorad). After imaging, the blot was stripped and re-probed using Beta Tubulin (BT7R) (Life Technologies MA5-16308-HRP) 1:10,000 as a loading control.

### Monitoring qPCR

MO-ChIP-exo library PCR amplification using Phusion High Fidelity DNA Polymerase (Thermo Scientific) was paused after 5 cycles in a subset of libraries. A 5-μL aliquot was removed from each library and used as template for qPCR. Library aliquots were amplified for another 20 cycles in an ABI Step One System adding SYBR green and 6-Carboxy-X-rhodamine (ROX) to the master mix as previously described (Buenrostro et al. 2013). A multicomponent plot (fluorescent signal vs cycle number) was obtained and used to determine the number of additional cycles necessary to reach optimal library concentration for sequencing. The number of cycles before the curve starts to increase exponentially was chosen as optimal. Libraries paused at 5 cycles were enriched for an additional 7 cycles, size selected, cleaned up, and analyzed in the Tape Station using High Sensitivity D5000 ScreenTape and Reagents. Completed libraries were quantified using the Library Quantification Kit, For Illumina (NEB) to confirm sufficient library for sequencing.

### pH monitoring

Changes in pH were monitored during crosslinking in multiple crosslinking and quenching conditions using an Orion Star A215 pH/Conductivity Benchtop Multiparameter Meter (Thermo Scientific). An aliquot ∼25 mL of media (Iscove's Modified Dulbecco's Medium (IMDM) + 10% fetal bovine serum (FBS)) or PBS was used to measure pH before and after adding formaldehyde to 1% final concentration. After 5 to 10 min a quencher was added, and the pH was remeasured.

### Sequencing

Completed libraries were pooled for each sequencing run based on their concentration and read allocation with a 1% PhiX spike-in, treated with Illumina's Free Adapter Blocking Reagent and cleaned up using Mag-Bind TotalPure NGS beads. Final pool concentration was determined through qPCR using NEB Next Library Quantification Kit, For Illumina in an ABI StepOne Plus Real-Time PCR System before diluting to 0.65 nM for loading. An Illumina NextSeq 2000 Sequencing System was used with a NextSeq 1000/2000 P2 XLEAP-SBS Reagent Kit (100 cycle) for paired-end sequencing with 50 bp each for read1 and read2.

### Data sources

Previously published K562 CTCF data from ChIP-exo 5.0 and ChIP-exo 1.1 protocols were sourced from GEO accession number GSE110681 (Rossi et al. 2018). Fastq files from these experiments were downloaded and processed using the same methods as data generated in this study.

### Data analysis

#### Read alignment

Adapter sequences were first trimmed from the paired-end fastq files using fastp (v.0.23.2) (Chen et al. 2018). Paired-end reads were aligned using BWA-MEM (v.0.7.17) (Li and Durbin 2010) with parameter -M against the human (hg38) or mouse (mm10) genomes. BAM files were sorted using samtools sort (v.1.16.1) (Li et al. 2009) and duplicate read pairs were marked using Picard MarkDuplicates (v.2.24.1) with arguments REMOVE_DUPLICATES = “false” ASSUME_SORTED = “true” DUPLICATE_SCORING_STRATEGY = “SUM_OF_BASE_QUALITIES” READ_NAME_REGEX = [a-zA-Z0-9] + :[0-9]:([0-9]+):([0-9]+):([0-9]+).*.” BAM files were then filtered to remove reads that are duplicated, unmapped, or not in proper pairs using samtools view (v.1.16.1) with arguments -h -b -f 0 × 3 -F 0 × 404.

#### Peak finding

Peaks for each individual ChIP-exo sample were determined using ChExMix (v0.52) (Yamada et al. 2018) using the following parameters: --threads 4 --expt ${expt} --ctrl ${ctrl} --format BAM --scalewin 1000 --noread2 --round 3 --minmodelupdateevents 50 --prlogconf −4 --alphascale 1.0 --betascale 0.05 --epsilonscale 0.2 --minroc 0.7 --minmodelupdaterefs 25 --pref −0.1 --numcomps 500 --win 250 --back ${back} --memepath ${memePath} --mememinw 6 --mememaxw 18 --seq ${genome} --exclude ${exclude} --q 0.01 --minfold 1.5.

Blacklist files (the ${exclude} argument) were sourced from ENCODE (Dunham, et al. 2012). Background files (the ${back} argument) are second-order Markov models created from the human or mouse genomes. Control samples (the ${ctrl} argument) represent pooled BAM files of all relevant control samples in each cell type, merged using samtools merge (v.1.16.1) (Li et al. 2009). This merger was performed across 13 K562 control samples (totaling 40,362,133 read pairs), 5 HepG2 control samples (13,990,398 read pairs), and 14 mES control samples (93,023,738 read pairs).

#### Fraction of reads in peaks scores

Fraction of reads in peaks (FRiP) scores for each sample are calculated by counting the fraction of mapped read 1 reads (ie the ChIP-exo resolution read) that intersect with peaks according to BEDTools intersect (v2.30.0) (Quinlan and Hall 2010). FRiP scores are typically calculated using peaks called from a given sample. However, the number of peaks called in each dataset depends not only on the quality of the ChIP experiment but also on other parameters such as sequencing depth and library complexity. To ensure that FRiP scores are a comparable metric of quality across samples, we use a common set of peaks for each transcription factor (TF) and cell type. The FRiP scores presented in the manuscript were defined with respect to peaks determined from ENCODE ChIP-seq data for each relevant TF and cell type (Dunham, et al. 2012). Specifically, we combined the following ENCODE narrowPeaks BED files using BEDTools sort and BEDTools merge (v2.30.0):

- K562 CTCF: ENCFF221SKA, ENCFF582SNT, ENCFF660GHM, ENCFF736NYC, ENCFF769AUF
- K562 USF1: ENCFF255QDL
- HepG2 CTCF: ENCFF119PKI, ENCFF199YFA, ENCFF205OKL, ENCFF240MUS, ENCFF664UGR
- mES CTCF: ENCFF052KVO, ENCFF533APC

We also tested an alternative strategy where peaks for each TF and cell type were determined by performing peak finding on merged sets of samples that each individually yield more than 1,000 peaks. This approach yielded FRiP scores that are highly correlated with FRiP scores from ENCODE-defined peaks (Pearson’s correlation coefficient >0.99 for each TF/cell type combination) and essentially unchanged relative rankings (Spearman’s rank correlation >0.99).

#### Estimated library size

We calculate the estimated complexity of a ChIP-exo DNA library (ie the number of unique DNA fragments in the initial library) using the same procedure as implemented in the EstimateLibraryComplexity function of the Picard Toolkit (2019). Specifically, this procedure is based on the following equation:


$$\frac{U}{C}=1-e^{\left(-\frac{N}{C}\right)},$$

where *C* is the number of distinct molecules in the library, *N* the number of paired-end reads (total mapped paired-end reads in our usage), and *U* the number of unique paired-end reads observed (number of deduplicated mapped paired-end reads in our usage). The value of *C* is solved using a bisection search. Note that this framing of the problem assumes that the sequencing process is an unbiased sampling with replacement, governed by a Poisson distribution. In reality, PCR and sequencing introduce substantial biases, so our library size estimates are unlikely to be highly accurate. However, the calculated estimated library size (ELS) values should be appropriate for comparing the relative library complexity across experimental conditions, as they are used here.

#### Poly-G read rates

Poly-G-containing reads are defined as those containing 10 or more consecutive Gs. The rates were calculated using an awk script applied to the fastq files.

#### Insert sizes

Read insert size distributions were calculated using Picard CollectInsertSizeMetrics (v.2.24.1) (Picard Toolkit 2019).

#### Differential binding analysis

To assess whether CTCF binding varied according to sonication levels, we compared CTCF ChIP-exo enrichment between samples prepared with 1 sonication cycle and 6 sonication cycles (Fig. 4b, Supplementary Fig. 3). The 6-cycle experiment was chosen because ChIP-exo signal is too weak in higher cycle experiments. ChIP-exo reads overlapping 200-bp windows centered on CTCF binding sites were counted for each replicate in each condition. EdgeR (v.4.4.2) (Robinson et al. 2010 ) was then used to normalize and compare enrichment levels across conditions.

## Results

### Overview of the ChIP-exo protocol

**Fig. 1.**
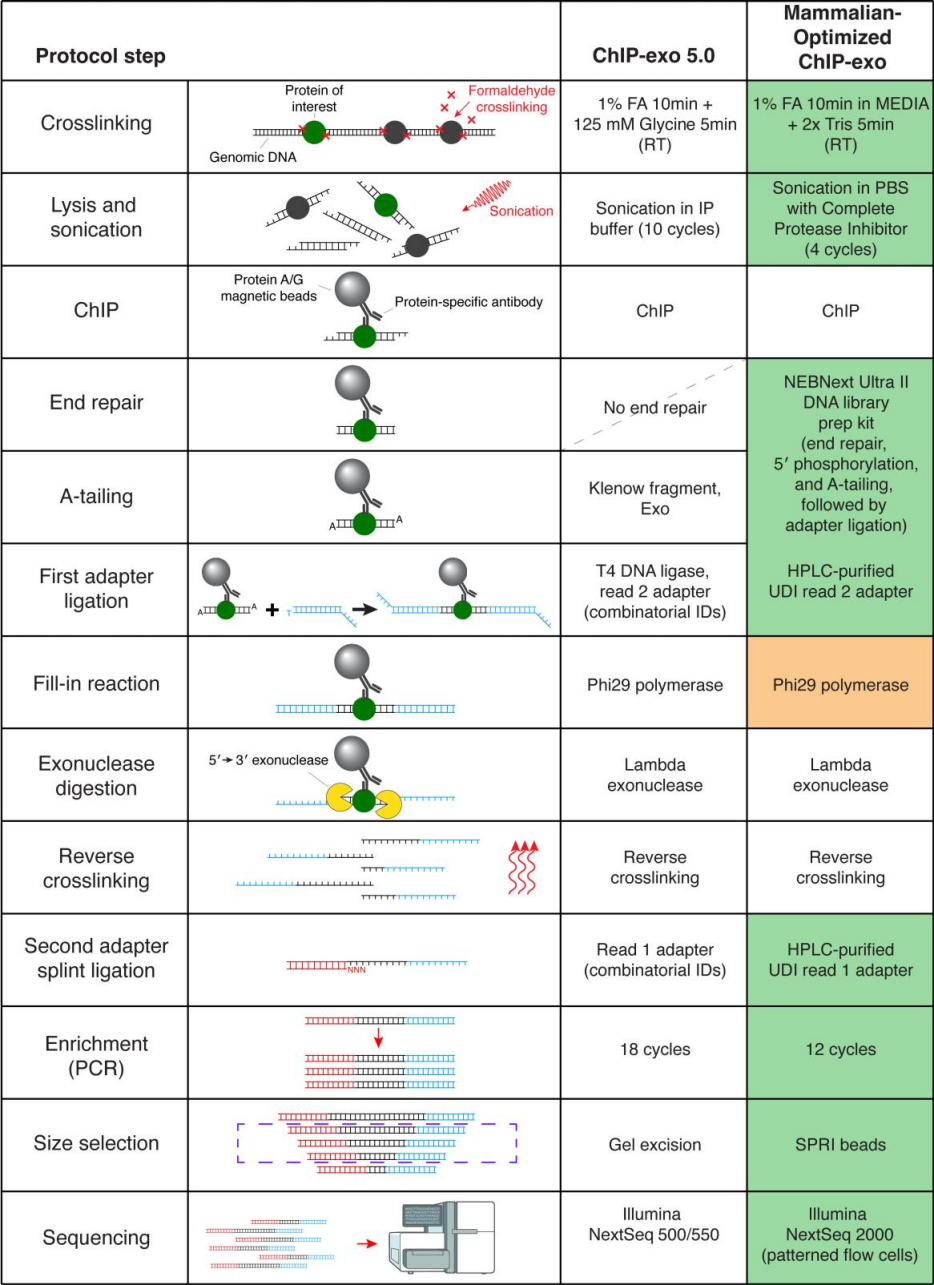
Summary of the ChIP-exo protocol, highlighting MO-ChIP-exo protocol modifications. Each row describes a key step in the ChIP-exo protocol. The second last column describes protocol steps in ChIP-exo 5.0, while the last column describes steps in MO-ChIP-exo. The shaded boxes in the last column highlight key protocol steps that were modified (green) or explored (orange) in MO-ChIP-exo.

The ChIP-exo 5.0 protocol offered a simplified series of steps compared with the original ChIP-exo protocol (Rossi et al. 2018; Fig. 1). In ChIP-exo 5.0, crosslinked cells undergo lysis and chromatin is sheared via sonication. The sheared chromatin is immunoprecipitated and directly subjected to A-tailing (in ChIP-exo 1.1 a polishing step preceded the A-tailing, but this was removed in ChIP-exo 5.0). The read 2 sequencing adapter is then ligated using the 3′ A overhang and a fill-in reaction fills in the 5′ overhang. Next, the defining step of ChIP-exo uses lambda exonuclease to digest single strands of DNA in the 5′ → 3′ direction until it is blocked by a crosslinked protein. Crosslinking is then reversed and the read 1 sequencing adapters are attached to the 5′ ends of the single-stranded DNA molecules via splint ligation. Adapter-bound fragments are enriched using PCR (18 cycles) and size selected (using gel excision) before sequencing.

Our initial attempts to execute the ChIP-exo 5.0 protocol on mammalian cells using a NextSeq 2000 sequencer identified several inefficiencies and some incompatibilities with newer patterned flow cell sequencing platforms. Specifically, we observed a high rate of “undetermined” reads that could not be assigned barcodes (17%). Among barcode-assigned reads, ∼16% contained poly-G sequences in read 2, limiting the use of read 2 for genome mapping. We also noted very high paired-end read duplication rates, reflecting low library complexity or potentially arising from the rapid clustering and amplification characteristic of the Illumina exclusion amplification technology. Overall, over 80% of sequenced reads were removed from analysis due to barcode ambiguity, de-duplication, or an inability to map to the genome. We were unable to find specific ChIP-exo peaks centered on cognate motifs in these initial libraries. We therefore set out to reevaluate various steps in the protocol with the goal of optimizing for mammalian cells and assessing the efficiencies of techniques that were unavailable when the ChIP-exo 5.0 protocol was defined. The highlighted cells in Fig. 1 summarize the protocol steps that we reassessed in this study, resulting in the MO-ChIP-exo protocol.

Our investigations primarily focus on ChIP-exo experiments targeting the CCCTC-binding factor (CTCF) in K562. CTCF is a highly conserved zinc finger protein best known as an insulator transcription factor that helps to establish chromosome architecture (Kim et al. 2015). CTCF offers a convenient target for quality assessments, given the availability of high-quality antibodies, its ubiquitous expression in mammalian cells, and the high conservation of CTCF binding sites across cell types (Chen et al. 2012). We use a series of quality metrics to compare sequenced ChIP-exo library quality across multiple conditions. FRiP, defined by ENCODE as the fraction of mapped reads that overlap peak regions, was used to assess ChIP enrichment and signal-to-noise (Landt et al. 2012). To facilitate comparisons of FRiP scores across samples, we measure it in relation to a common set of ENCODE CTCF peaks (see Materials and methods). ELS is an empirical estimate of the number of unique DNA fragments present in each sequencing library, thus serving as a library complexity measurement. Finally, the Peak Counts for each sample are reported by the ChExMix peak-finder for ChIP-exo data (Yamada et al. 2018, 2020) and represent the numbers of genomic regions that display a statistical enrichment of aligned reads in the ChIP-exo sample compared with a control.

### Optimizing crosslinking conditions for high-quality ChIP-exo

In vivo crosslinking, the first step in the ChIP-exo protocol, is critical for preserving protein–DNA interactions in a stable yet reversible manner. This reversibility is critical for isolating specific chromatin complexes of interest. While crosslinking is a pivotal step for ChIP, the ideal conditions have been debated for decades (reviewed in Sutherland et al. 2008; Hoffman et al. 2015). Optimal crosslinking balances the resolution of binding site detection with epitope loss from shearing. Because crosslinking efficiency is affected by the crosslinking agent as well as reaction pH, temperature, time, and concentration (Baranello et al. 2016; Ji et al. 2022; Xu et al. 2024), these conditions must be fine-tuned to detect biologically relevant interactions. Too little crosslinking will only capture the most robust interactions (Kasinathan et al. 2014), while too much crosslinking can fix even the most transient interactions, leading to insoluble complexes that mask or reduce epitope accessibility (Rozbeský et al. 2018).

Formaldehyde is the most widely used crosslinking agent, yet despite its widespread use, protocols vary in terms of the crosslinking vehicle (which may influence pH stability), temperature, formaldehyde concentration, and exposure time. The pH is a critical variable, as it affects both crosslinking reaction (Schroeder et al. 2019) and protein affinity (Akerström and Björck 1986; Watanabe et al. 2009). Buffers like PBS are commonly used to sustain a physiological pH during crosslinking (Keller et al. 2021; Park et al. 2025), but many studies fail to specify the vehicle used (Euskirchen et al. 2007; Johnson et al. 2007; Quon et al. 2023). Although practical from a technical standpoint, crosslinking cells in media introduce variations from the media components, which can alter pH or quench formaldehyde.

**Fig. 2.**
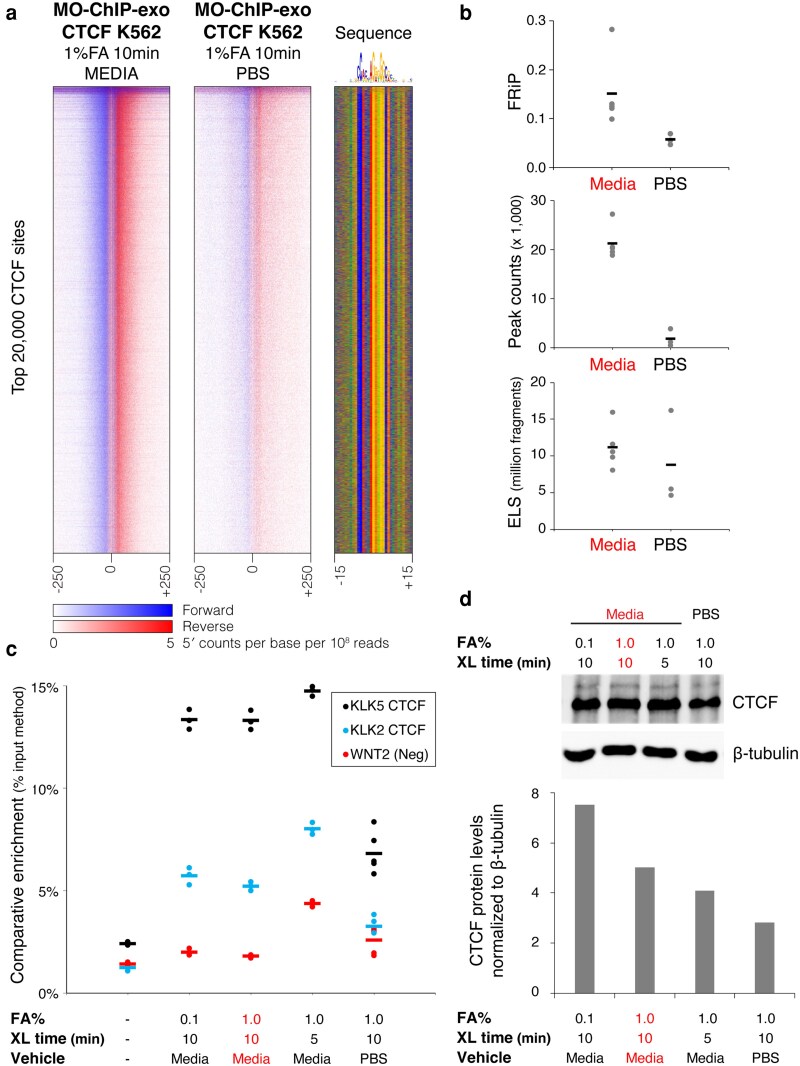
Crosslinking vehicle influences reaction efficiency and CTCF library quality. a) Heatmaps display normalized per base enrichment of 5′ read positions from ChIP-exo libraries constructed from cells crosslinked in media (*n* = 5, left) and PBS (*n* = 3, right). The heatmaps plot enrichment in 500-bp windows centered on the top 20,000 CTCF peaks (data merged across replicates for the purposes of heatmap display). Blue represents forward strand 5′ enrichment, while red represents reverse strand 5′ enrichment. b) Library quality metrics from ChIP-exo libraries constructed from cells crosslinked in media (*n* = 5) and PBS (*n* = 3), including FRiP scores, peak counts, and estimated library sizes. Each data point represents an individual ChIP-exo library and short lines represent the mean value across replicates. c) ChIP-qPCR was used to quantify comparative enrichment of CTCF at binding sites in the KLK locus (KLK2 and KLK5) and in an unbound negative control region (WNT2) across multiple crosslinking conditions. Dots represent individual technical replicates (*n* = 3 per condition, except *n* = 6 for KLK5 in PBS) and short lines represent mean values across replicates. d) Western blot (top panel) showing CTCF protein levels for K562 cells across multiple crosslinking conditions. And quantification of CTCF protein levels normalized to Beta tubulin (bottom panel) from the western blot. MO-ChIP-exo conditions are highlighted.

Our initial attempts to produce ChIP-exo libraries by crosslinking cells in PBS at room temperature (as recommended in ChIP-exo 5.0) resulted in poor quality metrics, including low FRiP scores, low library complexity, and low numbers of peaks (Fig. 2, a and b). We directly compared these libraries to libraries from cells crosslinked in rich media (IMDM + 10% FBS) and observed a striking improvement, with higher FRiP scores, larger ELS, and a greatly increased number of peaks.

To understand the differences observed between cells crosslinked in media and PBS, we examined the quality of the input chromatin. First, we compared the DNA yield for each condition after reverse crosslinking sonicated chromatin (ie before ChIP), finding a higher yield when cells were crosslinked in media (Supplementary Fig. 1a). This difference can be partly explained by the elimination of a centrifugation step needed to resuspend the cells in PBS, which affects yield by almost 50% without affecting viability (Supplementary Fig. 1, d to F). Interestingly, the fraction of DNA in the target fragment size range (100 to 500 bp) did not differ substantially between media and PBS conditions (Supplementary Fig. 1b). Next, we examined the CTCF-ChIPed material and determined that cells crosslinked in media yielded a significantly higher recovery of CTCF-bound DNA (Supplementary Fig. 1c). We also used ChIP-qPCR to quantify CTCF binding at 2 constitutive binding sites near the KLK2 and KLK5 gene loci (Khoury et al. 2020), compared with a locus at the WNT2 gene with no known CTCF binding. CTCF enrichment is significantly higher in the material collected from cells crosslinked in media compared with cells crosslinked in PBS (Fig. 2c) (1-tailed *t*-test, KLK2 *P* = 4.6 × 10^−5^, KLK5 *P* = 1.12 × 10^−6^). This enrichment was maintained even when formaldehyde concentration was decreased 10-fold or crosslinking time was halved (5 min instead of 10 min). Finally, we used western blots to assess the recovery of CTCF protein from K562 whole-cell lysates after crosslinking, finding that the amount of CTCF detected was higher for the samples crosslinked in media (Fig. 2d). Altogether, these results show that the crosslinking vehicle affects chromatin yield and potentially affects integrity of DNA–protein interactions or epitope accessibility, with crosslinking in media leading to improved ChIP quality and reduced background noise.

**Fig. 3.**
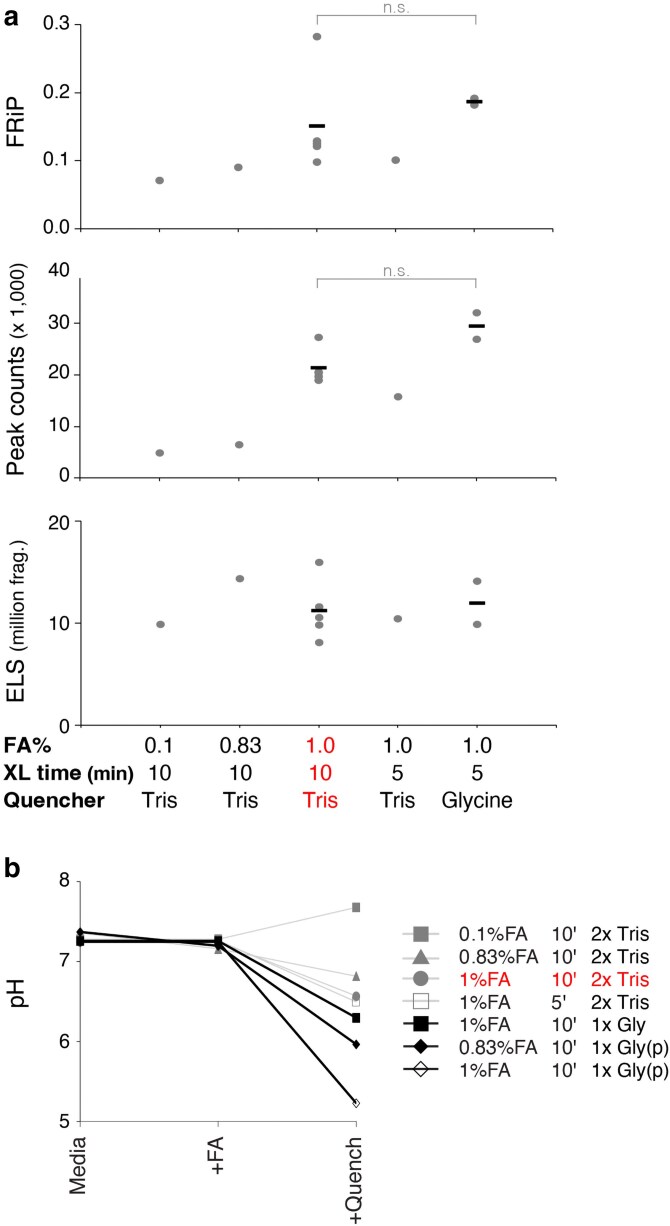
Formaldehyde concentration, time, and quenchers influence ChIP-exo library quality. a) Quality metrics are shown for CTCF libraries made under different crosslinking conditions in media using 0.1% (*n* = 1), 0.83% (*n* = 1), or 1% (*n* = 5) FA for 10 min, or 1% FA for 5 min using Tris (*n* = 1) or Glycine (*n* = 2) as quenchers. There is no significant difference between the indicated Tris and Glycine conditions (*t*-test; FRiP *P* = 0.17; peak counts *P* = 0.14). b) pH for media before and after crosslinking and quenching using multiple FA concentrations and times as well as different quenchers. MO-ChIP-exo conditions are highlighted.

After establishing media as the optimal crosslinking vehicle, we assessed other reaction variables. We found that a formaldehyde concentration of 1% yielded the best library quality, whereas 0.1% or 0.83% produced libraries with lower FRiP scores and low numbers of peaks (Fig. 3a). Reducing the crosslinking time from 10 to 5 min did not have a large effect (Fig. 3a). We also compared the quenching agents Tris and Glycine, which are added in excess to halt the crosslinking reaction. While Glycine is typically used to quench formaldehyde, Tris is a more efficient quencher given each molecule's ability to react with 2 formaldehyde molecules (reviewed in Hoffman et al. 2015). However, both quenchers produced libraries of comparably high quality (Fig. 3a). We noted that both quenchers, but particularly Glycine, lowered the media's pH. Specifically, the initial pH of the media (pH 6.9 to 7.3) was unchanged after adding formaldehyde but decreased to 5.2 to 6.3 after the addition of powder or liquid Glycine and to 6.5 to 6.7 after the addition of Tris (Fig. 3b). This pH drop may contribute to Glycine's effectiveness by slowing the crosslinking reaction rate. Despite their similar performance, we chose Tris for the MO-ChIP-exo protocol due to its greater theoretical quenching capability, which minimizes the risk of over-crosslinking. Finally, we confirmed that quenching conditions were unable to mitigate the detrimental effects of crosslinking in PBS (Supplementary Fig. 2).

Crosslinking immobilizes cell scaffold/topology, which eventually affects cell function and adhesion and inevitably leads to cell death. Fast crosslinking is expected to preserve interactions without immediate effects to viability, making crosslinking time a critical factor. Trypan blue staining confirmed that crosslinking as performed in MO-ChIP-exo (1% formaldehyde for 10 min, quenching with Tris for 5 min, and washing with cold PBS) does not immediately affect cell viability (Supplementary Fig. 1f) despite observations of adherent cells becoming dislodged in the presence of 1% formaldehyde with gentle shaking. Furthermore, to preserve chromatin integrity after quenching and to minimize the possibility of proteolysis, we introduce protease inhibitors during harvest and we snap freeze the cell pellets using liquid nitrogen. The liquid nitrogen snap freeze is conducive to cell lysis which is performed using modified Farnham lysis buffer with syringe passes to assist in mechanical disruption and followed by nuclear lysis in a RIPA variant buffer.

### Sonication conditions offer limited flexibility

Following the optimization of crosslinking, the next critical step is to establish sonication conditions that shear chromatin to an optimal size range. Efficient and consistent shearing can lead to higher reproducibility, as chromatin length has been shown to affect ChIP-seq quality and sensitivity (Keller et al. 2021). Over-sonication consistently reduces ChIP quality, particularly for transcription factors, while under-sonication can lead to a loss of signal for some targets. The ChIP-exo 5.0 protocol reported shearing chromatin in IP dilution buffer (containing 0.2% SDS) with a Diagenode Bioruptor for 10 cycles with 30 s On/Off intervals to obtain DNA fragments 100 to 500 bp in size. SDS is commonly used to aid sonication, yet it is known to interfere with antibody binding (Qualtiere et al. 1977). It is therefore common for chromatin sonicated in the presence of SDS to be diluted before proceeding to ChIP. To circumvent the use of SDS and to maintain a low volume ChIP reaction, we introduced a chromatin pelleting step and used PBS with protease inhibitors as the sonication buffer. We then systematically evaluated a range of sonication cycles (with 30 s On/Off intervals) using a Diagenode Bioruptor Pico.

**Fig. 4.**
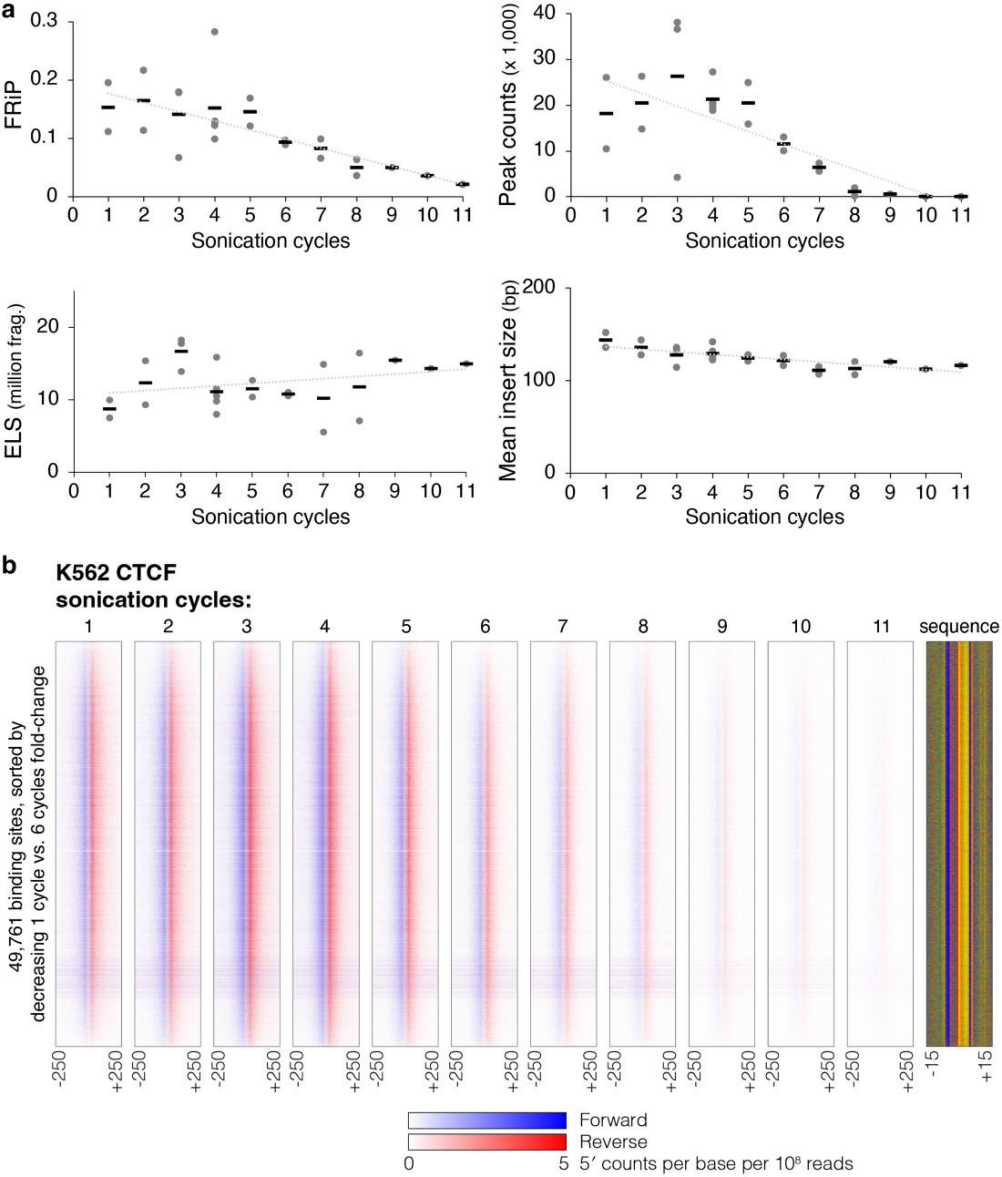
Sonication titrations and the effect of increased shearing on ChIP-exo library quality. a) Quality metrics (ie FRiP, peak counts, and ELS) and mean insert size (calculated from paired-end sequencing data) are shown for CTCF ChIP-exo libraries prepared with between 1 and 11 cycles of sonication (*n* = 2 for 1 to 3 cycles and 5 to 8 cycles; *n* = 5 for 4 cycles; *n* = 1 for 9 to 11 cycles). b) Heatmaps display normalized per base enrichment of 5′ read positions from CTCF ChIP-exo libraries prepared with between 1 and 11 cycles of sonication (data merged across replicates for the purposes of heatmap display). The heatmaps plot enrichment in 500-bp windows centered on all detected CTCF peaks. Blue represents forward strand 5′ enrichment, while red represents reverse strand 5′ enrichment. Binding sites are ordered according to decreasing fold-change enrichment between 1- and 6-cycle libraries.

As expected, both average fragment size and size distribution decrease with increasing sonication cycles (Supplementary Fig. 3) and this aligns with decreasing mean insert size between paired-end reads after sequencing (Fig. 4a). Surprisingly, ChIP-exo libraries present acceptable quality metrics after only a single cycle of sonication (Fig. 4a), even though a smaller proportion of fragments populate the target size range (ie 100 to 500 bp) compared with after 3 sonication cycles (Supplementary Fig. 3). Key metrics, including FRiP scores and the number of detected CTCF peaks, remained stable for libraries sonicated between 1 and 5 cycles but decreased sharply with further sonication, with almost no peaks detected after 8 cycles.

Interestingly, we found no variation in the identity of CTCF peaks that are detectable with low vs intermediate levels of sonication (Fig. 4b, Supplementary Fig. 4). We might expect that higher levels of sonication would “free up” chromatin associated with heterochromatic binding sites, thus leading to a difference in the cohorts of binding sites detected. However, no variation in CTCF enrichment levels beyond that expected between biological replicates was detected when comparing libraries with 1 and 6 cycles of sonication (Supplementary Fig. 4). Rather, a consistent cohort of sites displays ChIP-exo enrichment over a range of sonication cycles, and this signal gradually fades out as sonication cycles are increased (Fig. 4b). Thus, while chromatin length affects ChIP-exo quality and sensitivity, there is a range of sonication cycles that yields fragments of useful size and quality, without any impact on the cohorts of binding sites that are detected. Although a range of 2 to 5 sonication cycles produced high-quality libraries, we selected 4 cycles for our protocol due to the reduced variability in peak detection observed across multiple replicates.

### Revised library construction reduces sequencing artifacts on patterned flow cells

**Fig. 5.**
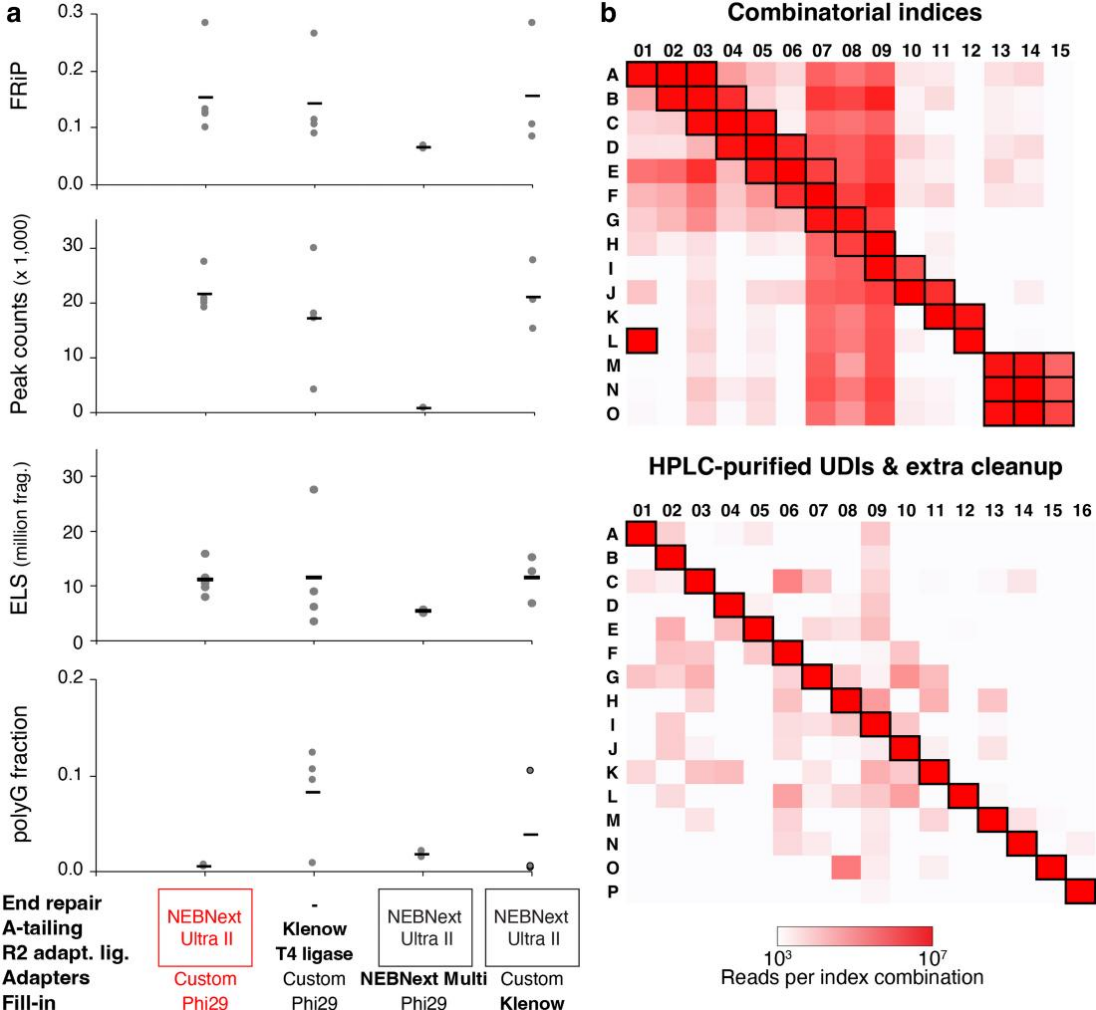
Exploring reaction alternatives confirms the advantage of using a kit to combine end repair, A-tailing, and first adapter ligation. a) Quality metrics are shown for CTCF libraries made using the NEBNext Ultra II DNA Library Prep Kit for the first adapter ligation (*n* = 5) compared with the custom A-tailing and ligations from ChIP-exo 5.0 (without end repair, *n* = 4), an attempt to use NEBNext Multiplex Oligo adapters (*n* = 2), and replacing Phi29 polymerase with Klenow for the fill-in reaction (*n* = 3). MO-ChIP-exo conditions are highlighted. b) Heatmaps summarize the numbers of observed sequencing reads assigned by demultiplexing to each possible combination of read 1 and read 2 indices across 2 NextSeq 2000 sequencing runs. In each panel, only index combinations whose square is highlighted by a black outline were included in the pooled sequencing library. The top panel summarizes a sequencing run that pooled 38 libraries with combinatorial indices. High read counts are observed outside the expected index combinations, indicating a high rate of index hopping. The bottom panel summarizes a sequencing run that pooled 16 libraries with HPLC-purified UDIs and an extra cleanup step after size selection.

Once sheared, chromatin is subjected to immunoprecipitation using an antibody conjugated to protein A/G magnetic beads, followed by sequential washes to prepare the material for library construction. The initial on-resin steps of library construction, where DNA fragments are prepared for sequencing adapter ligation, required significant modification to address issues arising from the use of an Illumina NextSeq 2000 patterned flow cell sequencer. The ChIP-exo 5.0 protocol involves directly A-tailing bead-bound chromatin fragments using a Klenow Fragment, followed by read 2 adapter ligation via TA ligation using T4 DNA ligase and a fill-in reaction using Phi29 polymerase. However, libraries prepared with this method exhibited a high proportion of read 2 sequences containing poly-G tracts (>10%) and a high rate of undetermined reads containing unexpected barcode combinations (Fig. 5, a and b). A noteworthy distinction in ChIP-exo is that read 1 sequencing will generate the precise DNA footprints that are characteristic of ChIP-exo's high resolution because read 1 sequencing adapters are added to the side of the molecule that was digested with exonuclease. Read 2 is the product of the first adapter ligation following A-tailing, and its positions are equivalent to a ChIP-seq experiment in resolution.

The high occurrence of poly-G artifacts, which result from the inability of the Illumina 2-color chemistry to distinguish between a string of “G” bases and “no signal,” suggested an inefficient read 2 adapter ligation reaction. The original ChIP-exo protocol included an end repair step which was deemed unnecessary in ChIP-exo 5.0 (Rossi et al. 2018). However, the change in sequencing technologies may have exacerbated an existing inefficient ligation issue. To address this, we replaced the custom A-tailing and ligation steps with the NEBNext Ultra II DNA Library Prep Kit for Illumina. This kit combines end repair and A-tailing into a single polishing reaction followed directly by ligation, streamlining the protocol. Switching to this kit greatly reduced read 2 poly-G artifacts while maintaining or slightly improving key quality metrics (Fig. 5a).

We explored additional modifications to the read 2 adapter ligation step, but these did not improve the results (Fig. 5a). First, we substituted the custom ChIP-exo adapters with NEBNext Multiplex Oligos. This change lowered the overall quality metrics and extended processing times due to the requirement to cleave the adapter's hairpin loop. Second, we tested an alternative enzyme for the fill-in reaction, which uses Phi29 polymerase to complete the read 2 adapter sequence. We hypothesized that the Klenow Fragment (3′ → 5′ exo-), which lacks exonuclease activity, might be more effective. However, no difference in library quality was observed between Phi29 and Klenow. Hence, the MO-ChIP-exo protocol retains the use of Phi29 for the fill-in reaction.

The second major issue was the high percentage of undetermined reads (averaging ∼17% of sequenced reads per library), which resulted from using the protocol's original combinatorial indexing strategy when pooled libraries were sequenced on a NextSeq 2000. Patterned flow cell sequencers are known to be more susceptible to index hopping, a phenomenon where reads from one sample are incorrectly assigned to another (Illumina 2018). Analysis of the undetermined reads revealed unexpected index combinations, consistent with index hopping (Fig. 5b, top panel). To mitigate this issue, we included a cleanup step using magnetic beads after size selection on the enriched libraries to completely remove any free adapters and possible adapter dimers before pooling libraries. We also switched to using unique dual indices (UDIs) that were purified via HPLC. ChIP-exo adapters are >50-bp long and incorporating HPLC purification removes truncated sequences that arise during synthesis. The use of UDIs enables bioinformatic filtering of hopped index combinations, which are then correctly classified as undetermined rather than being misassigned. This combination of changes greatly reduced the observed number of hopped indices and the proportion of undetermined reads overall (Fig. 5b).

Following these critical modifications to the initial read 2 adapter ligation steps, the remainder of the library construction, including the lambda exonuclease digestion and the read 1 adapter ligation, proceeds as described in the original ChIP-exo 5.0 protocol (Rossi et al. 2018).

### Refining input quantity, enrichment, and size selection to improve protocol performance

**Fig. 6.**
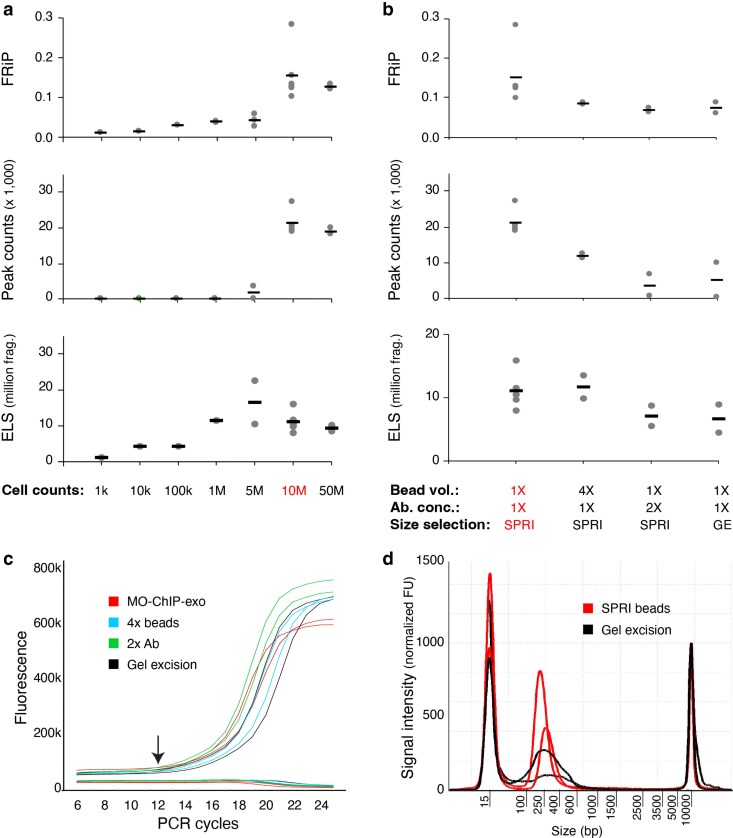
Exploring potential yield/quality improvements: input cell number, antibody and bead proportion, size selection, and enrichment. a) Quality metrics for CTCF ChIP-exo libraries when decreasing or increasing the input cell number (*n* = 1 for 1k, 10k, and 100k; *n* = 2 for 1, 5, and 50 M; *n* = 5 for 10 M). b) Quality metrics for CTCF MO-ChIP-exo libraries (*n* = 5) when increasing the protein A/G beads for antibody capture (*n* = 2), doubling the antibody concentration (*n* = 2), or using gel excision for library size selection as in ChIP-exo 5.0 (*n* = 2). (c) To monitor enrichment, a qPCR was performed using partially amplified libraries (5-cycles) as template, with 7 additional cycles showing sufficient enrichment for completed libraries. d) Electropherograms of completed CTCF libraries show a higher yield for libraries size selected with SPRI beads (*n* = 2) when compared with libraries size-selected using gel excision (*n* = 2). MO-ChIP-exo conditions are highlighted.

After addressing critical sequencing artifacts, we further refined the protocol to optimize library yield and quality. We first investigated the optimal input quantity. ChIP-exo 5.0 uses 10 M cells per library and reports high-quality yields from ∼250,000 cells and detection of CTCF binding signal from as few as 27,000 cells. We tested MO-ChIP-exo with a range of input cell numbers, from 1,000 cells to 50 million (Fig. 6a). While libraries with comparable complexity can be constructed from 1 and 5 M cells, their FRiP scores and peaks counts were greatly reduced when compared with the standard 10 M cells (Fig. 6a). Interestingly, increasing the input to 50 M cells did not improve any of the quality metrics, suggesting that there may be a component of the chromatin immunoprecipitation that is saturated or limiting the input material carried through. Neither increasing the bead volume 4-fold (from 50 to 200 μL) nor doubling the concentration of CTCF antibody resulted in improved quality metrics (Fig. 6b). These results confirmed that our MO-ChIP-exo protocol has achieved a robust balance between cell number, bead volume, and antibody concentration, with 10 M cells representing the optimal input.

Next, we sought to minimize PCR duplicates by optimizing the library enrichment step. The ChIP-exo 5.0 protocol recommends 18 cycles of PCR amplification, which we observed to produce an increase in sequence duplication rates and an excess of library material (average library concentration of 4 nM with some ranging as high as 18 nM). To avoid over-amplification, we implemented a qPCR-based monitoring strategy, similar to that used for ATAC-seq (Buenrostro et al. 2013). After an initial 5 cycles of PCR, a small aliquot (10%) of the library is monitored for an additional 20 cycles via qPCR using SYBR Green to precisely determine the number of additional cycles necessary to reach the target concentration without reaching saturation (where the target concentration is ∼0.5 nM to allow loading on the NextSeq 2000). This qPCR monitoring strategy reduced total amplification from the original 18 cycles to an average of 12 (ie initial 5 plus additional 7), thereby reducing PCR duplicates and increasing the proportion of unique fragments in each library while producing enough fragments for sequencing (average library concentration of 2 nM) (Fig. 6c).

Finally, we replaced the laborious and lossy gel excision step for size selection with a more efficient magnetic bead-based method. The ChIP-exo 5.0 protocol relied on gel excision (200 to 500 bp) to remove adapter dimers and to select library fragments of the desired length, but this process is delicate and can lead to sample loss. The ChIP-exo 5.0 protocol states that alternative size selection approaches lead to unacceptably high levels of adapter dimers (Rossi et al. 2018). However, we found that using SPRIselect DNA size selection magnetic beads (Beckman Coulter) for the size selection and cleanup step was faster and required less sample manipulation. Contrary to the original protocol's concerns, libraries purified with SPRI beads maintained or improved quality metrics, including lower duplication rates, compared with those purified by gel excision (Fig. 6, b to d). Thus, MO-ChIP-exo incorporates SPRI beads for size selection, in conjunction with TapeStation-based assessment of library fragment distributions. This change streamlined the workflow, reduced processing time, and enhanced overall library quality.

### Validation and application of MO-ChIP-exo

**Fig. 7.**
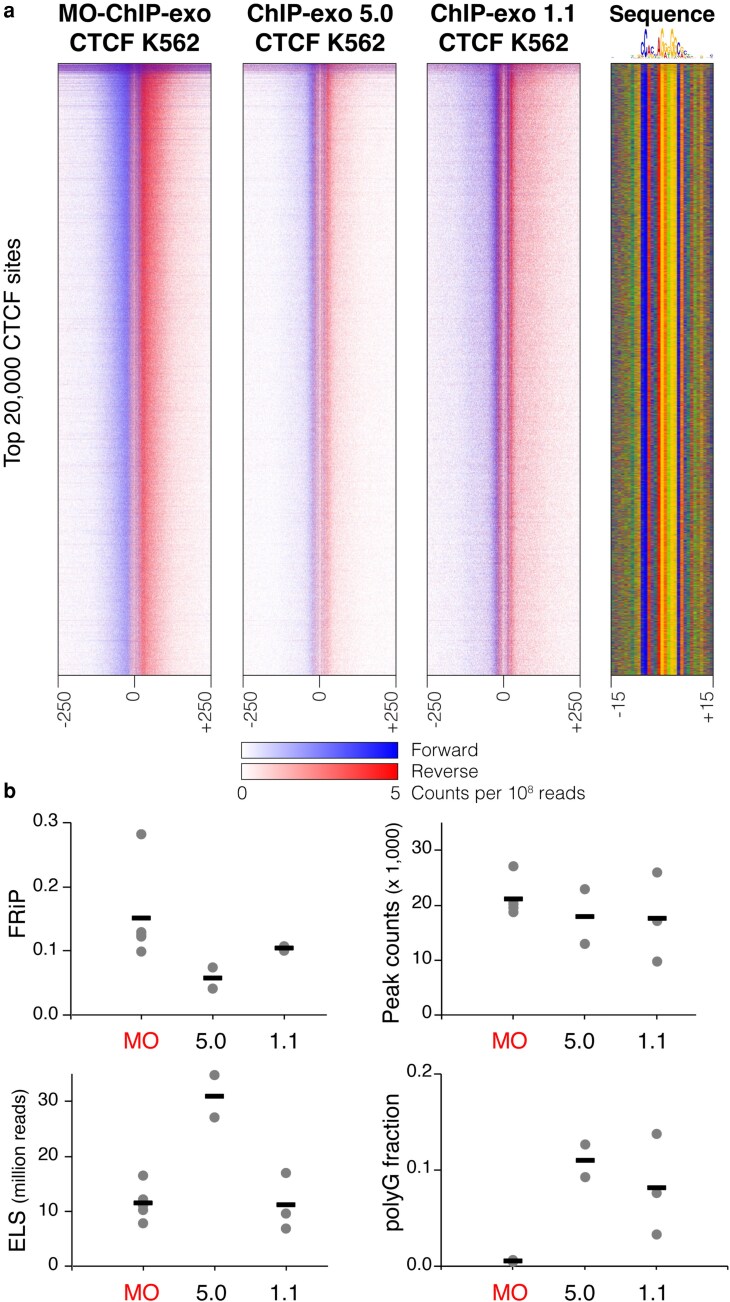
MO-ChIP-exo outperforms previous ChIP-exo protocols. a) MO-ChIP-exo data for CTCF in K562 cells (*n* = 2) compared with published K562 CTCF data from the simplified ChIP-exo 5.0 (*n* = 2) and the original ChIP-exo 1.1 (*n* = 3) protocols (Rossi et al. 2018). Heatmaps display normalized per base enrichment of 5′ read positions from each ChIP-exo protocol (data merged across replicates for the purposes of heatmap display). The heatmaps plot enrichment in 500-bp windows centered on the top 20,000 CTCF peaks. Blue represents forward strand 5′ enrichment, while red represents reverse strand 5′ enrichment. b) Quality metrics (ie FRiP, peak counts, and ELS) and percentages of read 2 that contain poly-G sequences are shown for MO-ChIP-exo, ChIP-exo 5.0, and ChIP-exo 1.1.

To validate the performance of MO-ChIP-exo, we compared our results to previously published K562 CTCF data from the original (ChIP-exo 1.1) and simplified (ChIP-exo 5.0) protocols, reanalyzing all datasets with our pipeline for consistency. MO-ChIP-exo libraries produce the highest FRiP scores and highest numbers of CTCF peaks, with a library complexity comparable to ChIP-exo 1.1 (Fig. 7). While ChIP-exo 5.0 libraries exhibited higher ELS scores, this was offset by lower FRiP scores and a significant proportion of poly-G reads (>10%), despite being sequenced in nonpatterned flow cells. Our results suggest that MO-ChIP-exo strikes a balance, preserving beneficial features from the original ChIP-exo 1.1 protocol, such as a polishing step, while integrating new optimizations that improve data quality and streamline the workflow.

**Fig. 8.**
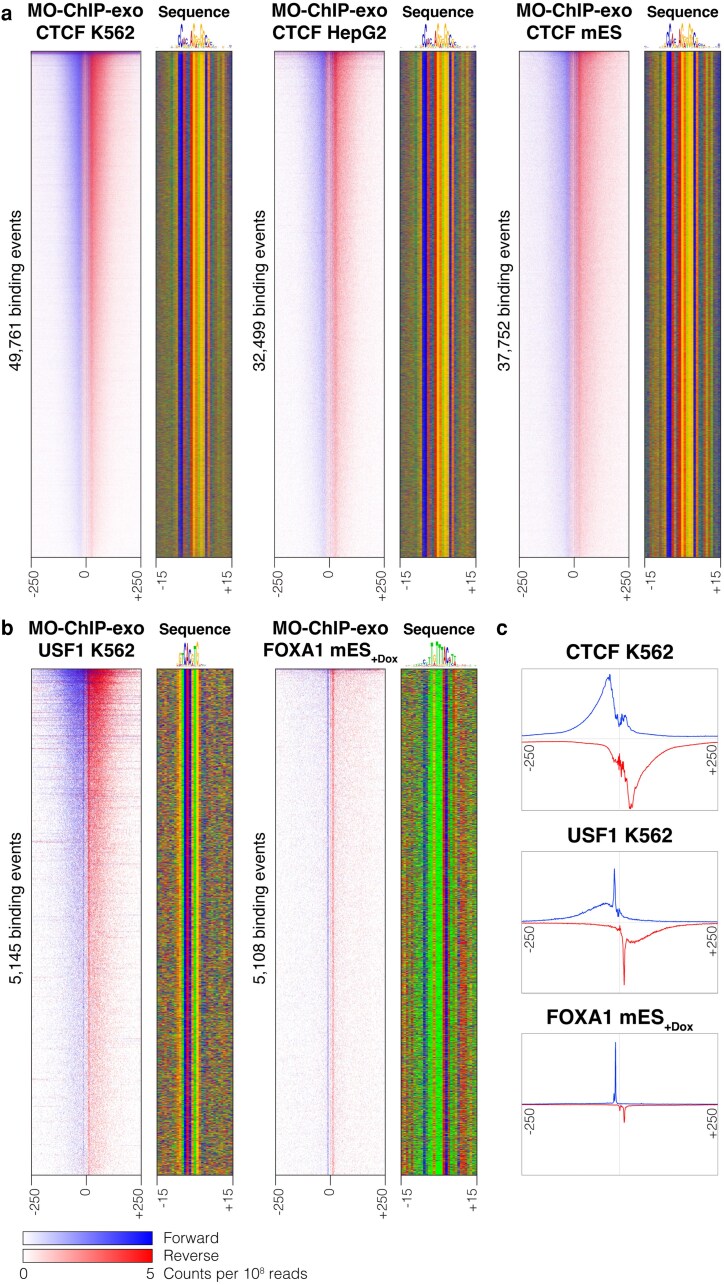
MO-ChIP-exo is applicable to additional cell types and protein targets. a) Heatmaps from MO-ChIP-exo experiments targeting CTCF in K562 cells (*n* = 5), HepG2 cells (*n* = 4), and mES cells (*n*= 4). Heatmaps display normalized per base enrichment of 5′ read positions from each ChIP-exo experiment (data merged across replicates for the purposes of heatmap display). The heatmaps plot enrichment in 500-bp windows centered on all CTCF peaks discovered in each cell type. Blue represents forward strand 5′ enrichment, while red represents reverse strand 5′ enrichment. b) Heatmaps (formatted as in a) targeting USF1 in K562 cells (*n* = 2) and FLAG-tagged FOXA1-DBD in mESC cells (*n* = 2). (c) Profile plots showing the average distribution of 5′ read positions (blue = forward strand, red = reverse strand) from MO-ChIP-exo experiments targeting CTCF (K562), USF1 (K562), and FOXA1-DBD (mES). The profiles show relative enrichment in 500-bp windows centered on all peaks discovered in each experiment.

To demonstrate the broader applicability of MO-ChIP-exo, we applied it to characterize CTCF binding in 2 adherent mammalian cell lines: human HepG2 and mouse embryonic stem cells (mESCs). Both cell lines were trypsinized, quenched, and resuspended in fresh media for crosslinking. Cell counts were obtained at this point to mitigate the effect of cell loss from additional centrifugation steps. While we performed sonication titrations for both cell lines, we determined that the optimal setting of 4 sonication cycles based on K562 cells was also appropriate for these cell lines (Supplementary Fig. 3). MO-ChIP-exo produced high-quality CTCF ChIP-exo libraries in both cell lines, with quality metrics comparable to the K562 libraries (Fig. 8a).

Finally, to demonstrate that MO-ChIP-exo's improved performance is not limited to experiments targeting CTCF, we applied the protocol to survey genome-wide binding of 2 additional protein targets (Fig. 8b). We first applied MO-ChIP-exo to the upstream transcription factor 1 (USF1), a basic helix–loop–helix leucine zipper family transcription factor that is constitutively expressed in most tissues (Pajukanta et al. 2004). MO-ChIP-exo experiments targeting USF1 in K562 cells find over 5,000 binding events, which are almost exclusively centered on USF1's cognate E-box motif (Fig. 8b). To confirm the effect of crosslinking vehicle observed for CTCF on an additional protein target, we compared USF1 MO-ChIP-exo experiments prepared by crosslinking cells in media and PBS. Per-replicate FRiP scores and peak counts were substantially higher for USF1 MO-ChIP-exo samples crosslinked in media compared with those crosslinked in PBS, confirming the advantages of crosslinking in media (Supplementary Fig. 5). Second, we applied MO-ChIP-exo to the DBD of Forkhead Box Protein A1 (FOXA1), a transcription factor that plays crucial roles in development (Lee et al. 2005). We expressed an inducible, FLAG-tagged FOXA1-DBD in mouse embryonic stem cells which do not endogenously express FOXA1, using an induction system described previously (Iacovino et al. 2011). MO-ChIP-exo using an antibody targeting the FLAG epitope tag finds over 5,000 binding events centered on FOXA1's cognate motif, demonstrating the feasibility of MO-ChIP-exo for characterizing epitope-tagged transcription factors (Fig. 8b). We note that the shape of MO-ChIP-exo 5′ read distributions at peaks vary according to the crosslinking pattern induced by the targeted protein, with the FOXA1-DBD displaying a narrow, punctate pattern (Fig. 8c). Variation in crosslinking patterns is a distinguishing feature of ChIP-exo experiments and has been used by us and others to characterize protein–DNA binding modes (Yamada et al. 2018). Overall, our data demonstrate that the MO-ChIP-exo protocol is versatile and not limited to high-abundance protein targets such as CTCF.

## Discussion

While ChIP-exo offers unparalleled resolution in mapping protein–DNA interactions, the protocol's complexity has limited its widespread adoption. This study presents MO-ChIP-exo, a version of the ChIP-exo protocol that is systematically optimized for use in mammalian cells and adapted for compatibility with patterned flow cell sequencing platforms. The main differences between MO-ChIP-exo and the previous ChIP-exo 5.0 protocol are summarized in Fig. 1. Our work addresses critical, often unreported, methodological variables and introduces modifications that enhance data quality, workflow efficiency, and overall reliability.

One important consideration that has emerged from our results is the crosslinking vehicle. We demonstrate that crosslinking cells directly in their growth media is superior to using PBS. Crosslinking in PBS resulted in lower library quality, fewer detected peaks, and reduced yield of both DNA and the target protein. Our data suggest that this effect is partially due to the loss of cells during the additional centrifugation step to replace media with PBS. The loss of cells can be proportionally minimized if cell harvesting is performed in bulk, but this is not always possible due to cost or sample collection constraints. While using media introduces variability from its components, it avoids sample loss and, in our hands, proved essential for generating high-quality input material. This highlights the importance of empirically determining optimal conditions, as even seemingly minor procedural details can profoundly affect results.

We further optimized the crosslinking reaction itself. Our results show that 1% formaldehyde for 10 min produces suitable crosslinking, aligning with other ChIP-seq and ChIP-exo protocols. We elected to use 2-fold molar excess of Tris as a quencher instead of Glycine. Although both are effective, Tris inherently has a greater quenching capacity. We also observed that Glycine significantly lowers the pH of the reaction, which likely contributes to its efficacy by slowing the crosslinking rate. The importance of pH in fixation has been explored for immunofluorescence (Berod et al. 1981), showing that a higher pH achieved optimal fixation. This is in line with our observation that pH may play a part regulating the crosslinking quench. We note that for a robust and abundant target such as CTCF, a range of crosslinking conditions may be successful. More stringent crosslinking conditions may be needed for successful detection of less abundant or weaker-binding factors, and we recommend that crosslinking parameters be carefully re-optimized for new targets or epitopes.

Consistent chromatin shearing is also critical, especially for patterned flow cell sequencers such as the Illumina NextSeq 2000. These platforms use exclusion amplification, a clustering method that exhibits a bias toward smaller DNA fragments. It is therefore important to balance the generation of smaller fragments, which improves clustering efficiency, with the ability (later in the protocol) to efficiently remove adapter dimers that can otherwise dominate the sequencing run. ChIP-seq and ChIP-exo protocols typically aim to select DNA fragments between 100 and 500 bp in length. Our sonication titrations show that a high proportion of DNA fragments will fall in this optimal size range after 1 to 5 sonication cycles. We selected 4 cycles as the standard for MO-ChIP-exo, as it consistently produced the highest number of peaks with low replicate variability. This number of cycles also provided consistent results across several cell lines.

The most significant modifications in our protocol were driven by the need to resolve sequencing artifacts specific to patterned flow cells. Our initial libraries were plagued by a high rate of index hopping and the presence of poly-G tracts in read 2. We addressed index hopping by introducing a magnetic bead-based cleanup step after size selection to remove free adapters. We also replaced the original combinatorial indices with HPLC-purified UDIs, which allow for the bioinformatic removal of misassigned reads. The poly-G artifact, which pointed to an inefficient first adapter ligation, was resolved by re-introducing an end repair step. By incorporating a modern library preparation kit, we consolidated the end repair, A-tailing, and ligation reactions, which not only fixed the ligation issue but also streamlined the protocol by eliminating intermediate washes.

Finally, we refined several steps to improve workflow efficiency and yield. We confirmed that samples with 10 million cells provide the optimal input, as lower cell numbers reduced data quality and higher cell numbers offered no benefit. To minimize PCR duplicates, we replaced the fixed-cycle enrichment with a qPCR-based monitoring approach that significantly reduces total library amplification. Furthermore, we substituted the gel excision step for size selection with a faster and more efficient magnetic bead-based method, which improved DNA recovery and lowered duplication rates.

ChIP-exo is a laborious protocol with numerous steps that are critical to overall success. A major challenge is the lack of informative quality control checkpoints that assess the efficiency of enzymatic reactions, making troubleshooting very difficult before sequencing. ChIP-exo's signature exonuclease step degrades about half of an already limited amount of starting material. While we recommend integrating the use of a fragment analyzer to assess library quality and fragment size distribution, these DNA library metrics are not predictive of the success of the ChIP reaction. In our investigations, for example, estimated library size was not a good predictor of FRiP score or peak counts (Supplementary Fig. 6). The MO-ChIP-exo protocol was designed to navigate these difficulties, providing a more robust and reliable path to generating high-quality libraries. Combined with the critical updates for sequencer compatibility, our improvements result in a validated protocol that produces libraries with superior FRiP scores and peak counts compared with previous ChIP-exo protocols. MO-ChIP-exo has been successfully applied to different cell types and alternative transcription factors, demonstrating its versatility.

## Supplementary Material

jkaf270_Supplementary_Data

## Data Availability

All raw and processed sequencing data produced in this work are available from GEO accession number GSE305217. Supplemental material available at G3 online.
